# Fine-Scale Monitoring of Industrial Land and Its Intra-Structure Using Remote Sensing Images and POIs in the Hangzhou Bay Urban Agglomeration, China

**DOI:** 10.3390/ijerph20010226

**Published:** 2022-12-23

**Authors:** Lingyan Huang, Shanshan Xiang, Jianzhuang Zheng

**Affiliations:** 1 Business College, Zhejiang University City College, Hangzhou 310015, China; 2 Institute of Agricultural Remote Sensing and Information Technology, College of Environmental and Resource Sciences, Zhejiang University, Hangzhou 310058, China

**Keywords:** industrial land, industrial intra-structure, urban agglomeration, points of interest, Google Earth

## Abstract

China has experienced rapid industrial land growth over last three decades, which has brought about diverse social and environmental issues. Hence, it is extremely significant to monitor industrial land and intra-structure dynamics for industrial land management and industry transformation, but it is still a challenging task to effectively distinguish the internal structure of industrial land at a fine scale. In this study, we proposed a new framework for sensing the industrial land and intra-structure across the urban agglomeration around Hangzhou Bay (UAHB) during 2010–2015 through data on points of interest (POIs) and Google Earth (GE) images. The industrial intra-structure was identified via an analysis of industrial POI text information by employing natural language processing and four different machine learning algorithms, and the industrial parcels were photo-interpreted based on Google Earth. Moreover, the spatial pattern of the industrial land and intra-structure was characterized using kernel density estimation. The classification results showed that among the four models, the support vector machine (SVM) achieved the best predictive ability with an overall accuracy of 84.5%. It was found that the UAHB contains a huge amount of industrial land: the total area of industrial land rose from 112,766.9 ha in 2010 to 132,124.2 ha in 2015. Scores of industrial clusters have occurred in the urban-rural fringes and the coastal zone. The intra-structure was mostly traditional labor-intensive industry, and each city had formed own industrial characteristics. New industries such as the electronic information industry are highly encouraged to build in the core city of Hangzhou and the subcore city of Ningbo. Furthermore, the industrial renewal projects were also found particularly in the core area of each city in the UAHB. The integration of POIs and GE images enabled us to map industrial land use at high spatial resolution on a large scale. Our findings can provide a detailed industrial spatial layout and enable us to better understand the process of urban industrial dynamics, thus highlighting the implications for sustainable industrial land management and policy making at the urban-agglomeration level.

## 1. Introduction

Since the implementation of the Open Door Policy in 1978, China has witnessed unprecedented rates of urbanization and industrialization. Despite the huge economic benefits brought about by this rapid urbanization and industrialization, severe problems have arisen such as the loss of high-quality cultivated land [1,2], environmental pollution [3,4,5], and ecological degradation [6]. Industrial land use is one of the most dominant urban land-use categories in most Chinese cities. However, due to inefficient supervision and management, the industrial land utilization in China is of low efficiency, and the proportion of industrial land to built-up land is apparently much larger compared with that of developed countries. It was reported that the industrial land accounted for over 20% of the total built-up land in Zhejiang [7], a fast-growing province in eastern China, whereas the proportions in those famous industrial megacities such as Chicago and Detroit are maintained at a level of 5–10% [8].

Faced with the continuous demand for high-quality urban development and sustainable industrial upgrading, a series of policies and plans were implemented that were aimed at optimizing the industrial structure. For instance, in 2008, the State Council of China formally announced that it would scientifically plan industrial land and promote industrial land-use efficiency, especially in the development zones. Manufacturing of China (2025)*,* which was proposed in 2015, was the first action agenda to execute the Manufacturing Power Strategy. The agenda highlighted the inevitable necessity of developing advance industries and cultivating competitive industrial clusters. In summary, the industrial land development toward sustainability and high quality has become an essential part and inevitable course for China.

In this context, metropolises in China have experienced a profound transformation from traditional industries to modern financial and business services [9]. Manufacturing industries has been requested to move toward the urban fringes due to intensive urban land use [10]. Meanwhile, these industries are also undergoing structural optimization based on the comprehensive consideration of economic benefits, technology innovation, and environmental friendliness of the industries. The abovementioned industrial transformation has led to the drastic changes in the industrial land and its intra-structure from the spatial perspective. Monitoring the spatial distribution of industrial land and identifying the industrial intra-structure accurately and duly at a fine scale is the important first step in industrial land management. Sensing where industry is currently expanding or migrating and in what particular functions can help government officials to supervise informal industrial land use, evaluate industrial use efficiency, and enact integrated development policies [11,12].

With the rapid development of remote sensing technologies, a series of high-resolution remote sensing images have emerged that provide abundant information for urban land classification and industrial land mapping [13]. Numerus studies have focused on urban land classification based on object-oriented classification and deep learning algorithms by mining multiple features such as shapes, textures, spectral information, and spatial context information [14,15,16]. Nevertheless, it is still a challenging task to effectively distinguish industrial land from other urban land such as residential land and commercial land because remote sensing data do not provide sufficient social function information. Furthermore, it is difficult to identify the intra-structure of industrial land. So far, cadastral survey data are considered the most favorable data theoretically. However, these data are not allowed to be released to the public in China [17].

In the past few years, online volunteered geographic information (VGI) has emerged as a new data source that makes it possible to capture urban land use dynamics at a finer level [18]. One promising type of VGI for our purpose is point of interest (POI) data. Currently, there are scores of voluntarily generated POI directories on map service platforms such as Google, Baidu, and Gaode. Each POI is the abstract expression of a geographical entity and includes a name, coordinates, tags, etc. Efforts have been made to use POI data to delineate urban function areas [19,20], commercial hotspots [21], and urban boundaries [22]. Several studies have used the emerging frequency of different types of POI data to identify the urban land uses at the block level [23]. Yao et al. introduced a natural language processing (NLP) method and used a Word2Vec model to mine semantic information for urban land-use classification on the basis of the characteristics of POI data [24]. By integrating NLP and a machine learning (ML) algorithm, Jia et al. successfully classified different industries based on industrial POI data [25]. These studies indicated that POIs have a great potential to characterize the different land-use functions and industrial structure. However, based on the existing literature, we have little quantitative information on the internal structure of industrial land at a fine parcel level combined with a lack of an effective methodology to identify industrial intra-structure. Such information is crucial for the site selection of industries and identification of ecological pressure areas, thereby helping decision-making authorities manage manufacturing industries and achieve sustainable environment. In addition, previous studies have focused on the typical economic development zone or the single-city scale [26,27], but scant attention has been paid to the urban agglomeration scale. Urban agglomeration has become the new region unit in global competition and international division [28]. A more reasonable and sustainable mode of industrial land allocation and structure optimization is crucial for promoting urban-agglomeration development and competitiveness [29]. Hence, to take the important first step, studies on monitoring industrial land and the internal structure dynamics of urban agglomerations in developing countries with special national conditions are urgently needed, especially in China.

This paper aimed to develop a novel framework to monitor industrial land change and its internal structure and to examine how industrial land expands, regenerates, and upgrades in the urban agglomeration of China during the industrial transformation era. The urban agglomeration around Hangzhou Bay was selected as the case study area. The process of industrial land change in Hangzhou Bay involves both expansion and renewal. Specifically, the expansion represents the non-construction land such as cultivated land, green spaces, and water bodies converted into industrial land. The renewal primarily involves industrial brownfields being redeveloped for multiple land uses such as residential land, commercial land, green spaces, and other urban infrastructure [30]. The specific objectives were to: (1) develop a framework for identifying industrial land and intra-structure based on POI data and Google Earth (GE) images using NLP and ML to create a detailed and accessible database of industrial land use; (2) apply spatial analysis tools to characterize the spatial industrial land dynamics and to measure the uneven distribution of different industrial structures; (3) unveil the characteristics of the industrial expansion and renewal process; and (4) provide detailed information and scientific references for industrial land management in the future.

## 2. Materials and Methods

### 2.1. Materials

#### 2.1.1. Study Area

**Figure 1:**
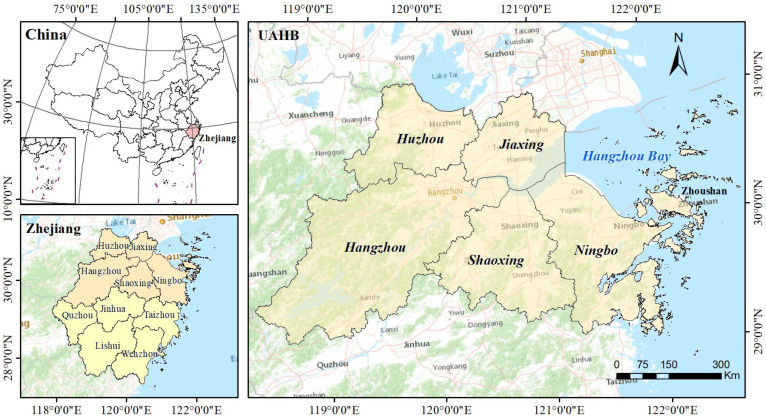
Location of the UAHB in Zhejiang Province, China.

The urban agglomeration around Hangzhou Bay (UAHB) is located in northern Zhejiang Province and southeastern Shanghai (118°20′–123°25′ E, 29°13′–31°11′ N) (Figure 1). The UAHB includes six large cities (Hangzhou, Ningbo, Jiaxing, Huzhou, Shaoxing, and Zhoushan). It had an area of 45,400 km^2^ and a total population of 24.9 million in 2015. In the past three decades, the UAHB has been experiencing rapid urbanization and economic development. The gross domestic product (GDP) of the UAHB increased from RMB 8.6 billion in 1978 to RMB 2929.0 billion in 2015, which accounted for 63% of Zhejiang Province. In Zhejiang Province, the regional block economy is the dominant industrial form and has become a powerful engine to accelerate regional economic growth.

Due to the influences of natural conditions, resource distribution, and policy guidance, there have been obvious regional divergences of industrial structure types between different cities across the UAHB. The industrial structure of each city has formed with local characteristics. For instance, the textile industry is a traditional pillar industry in the cities of Jiaxing, Huzhou, and Shaoxing; the logistics, advanced equipment, and household appliance industries play important roles in Ningbo; the logistics industry also plays a significant role in Zhoushan; and Hangzhou has become famous for the electronic information industry in recent years.

In 2003, the “Urban agglomeration space developing strategy planning around Hangzhou Bay, Zhejiang Province” was released. The planning firstly announced the mission of the UAHB Industrial Belt construction. Furthermore, “Powerful Zhejiang province construction depending on industry planning” and “New urbanization development planning in Zhejiang Province” were published in 2012. Both of them stressed that more attention should be paid to exploring the mechanism for industrial belt development across the UAHB. All of these circumstances indicate that the UAHB has been regarded as a pilot area by the central government to implement economic industrial transformation and measures for upgrading. Therefore, the UAHB presents a typical study area for industrial land and intra-structure monitoring.

#### 2.1.2. Data Preparation

The detailed datasets used in this study were as follows. (1) The POI datasets were obtained from application programming interfaces (APIs) provided by Google Maps. The Google POIs included diversified types of POIs such as education, hotel, entertainment, government, residential community, industrial company, etc. We selected POIs of industrial companies for our purpose, which resulted in 63,236 records from 2010 and 100,892 records from 2015. Each industrial POI had six attribute values: CITYCODE, COMPANY NAME, ADDRESS, TEL, X-coordinates, and Y-coordinates. The quality of industrial POIs was validated by checking 200 randomly sampled sites manually; the accuracy level was 97.8%. Other types of POIs including education, hotel, entertainment, government, and residential community were also used to help identify industrial renewal processes. (2) High-resolution Google Earth (GE) images at a spatial resolution of 1.03 m on 31 December 2010 and 31 December 2015 from the Google Earth Engine were used for manual photointerpretation of industrial land. The images were orthorectified into the Universal Transverse Mercator (UTM) projection system. For a cloud-free atmospheric condition in an entire image, there was no need for atmospheric correction in the preprocessing step. (3) The road networks included national roads, provincial roads, primary highways, railways, county roads, urban roads, and other small roads.

### 2.2. Methods

**Figure 2:**
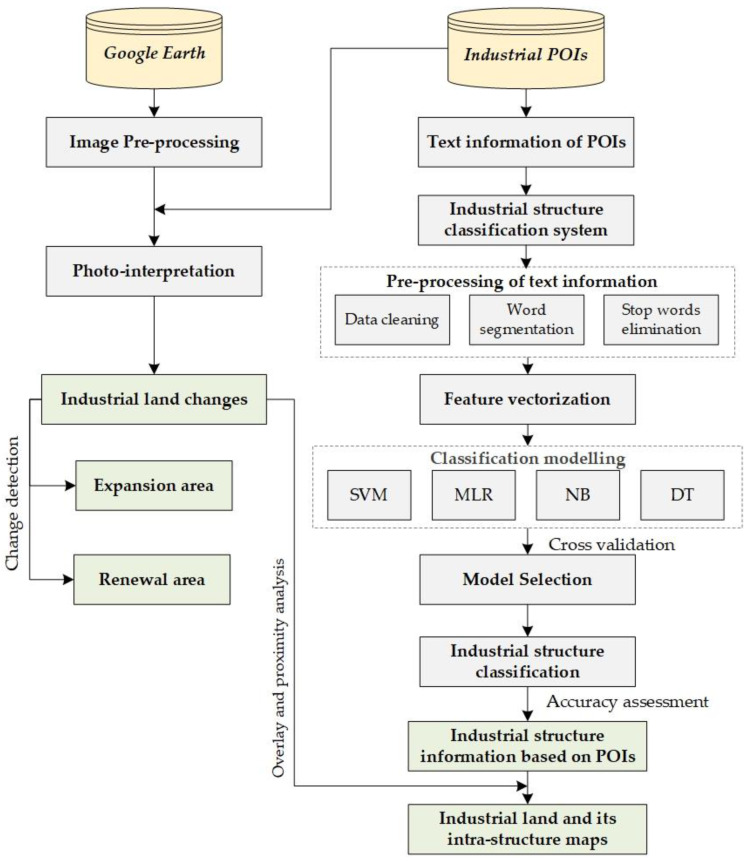
Flowchart of the methodology.

The framework used for this study consisted of three stages as shown in Figure 2: (1) identifying industrial intra-structure based on industrial POIs using NLP and ML; (2) industrial land mapping and change detection (i.e., industrial expansion and renewal) using Google Earth Maps; and (3) kernel density estimation of industrial land and its intra-structure.

#### 2.2.1. Identifying Industrial Structure Using Natural Language Learning and Machine Learning

Based on the latest “National Economic Industry Classification Criterion (GB/T4754-2011)” regulation and the industrial characteristics of the UAHB, the industrial structure classes were aggregated into 19 subclasses that fell into three groups of first classes (Table 1).

In this section, the main three steps in identifying industrial structures based primarily on the enterprise name were: (1) word segmentation; (2) feature vectorization; and (3) classification modeling. Firstly, in terms of word segmentation, the text information (enterprise name) of each industrial POI was divided into words using an open-access tool for Chinese text segmentation called Jieba. Based on prefix dictionaries, Jieba scans the text efficiently and generates a directed acyclic graph of all possible Chinese word phases (https://github.com/fxsjy/jieba, accessed on 17 June 2022). It uses dynamic programming to search the maximum probability path and obtains the most proper groups based on word frequency [31,32]. Furthermore, with the integrated hidden Markov model and Viterbi algorithm in Jieba, unknown words can be easily handled [33]. Examples of industrial text segmentation results are shown in Table 2. The Chinese company names were all translated into English. The accuracy of segmentation processing was 90.5%.

**Figure 3:**
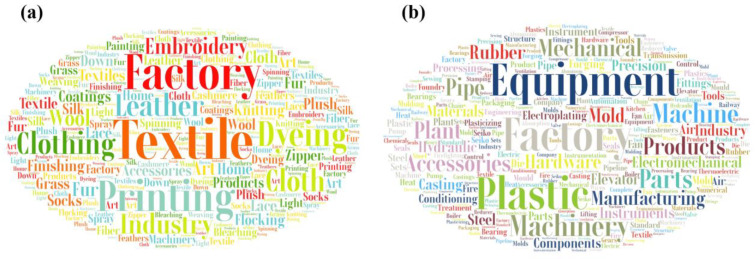
Related keyword examples for different industrial structures: (**a**) textile and clothing manufacturing; (**b**) equipment manufacturing.

Secondly, the feature vectorization consisted of the feature extraction and the feature selection. The feature extraction was required to eliminate numbers, punctuation, personal names, and other irrelevant information from the words relating to industrial structure. The left words were regarded as a keyword dictionary (Figure 3), and then we presented the text in vector form that could be the evidence for industrial structure classification. Feature selection led to improved classification efficiency and reduces the computational complexity. A classical algorithm; namely, the term frequency–inverse document frequency (TF-IDF), was applied for feature selection and included term frequency (TF) and inverse document frequency (IDF). TF is the frequency of words in text, and IDF reflects the distribution of feature items in the entire corpus. TF-IDF determined the weight for each word in the enterprise name. The TF-IDF was calculated as:

(1) $$W_{dt}=\frac{tf_{dt}\times \mathrm{lg}\left(\frac{N}{n}+0.01\right)}{\sqrt{\sum_{p=1}^{K}\left[tf_{dt}\times \mathrm{lg}\left(\frac{N}{n}+0.01\right)\right]}}$$

where t represents the word, d represents the text of enterprise name, $W_{dt}$ represents the weight of t in d, $tf_{dt}$ represents the frequency of t appearing in d, N represents the total number of d in the corpus, n represents the number of texts that contain t in the corpus, and K represents the number of t in d. The formula shows that the TF-IDF was proportional to the frequency of a word in the document and inversely proportional to the frequency of it in the corpus.

Thirdly, the classification models considered were the multivariate logistic regression (MLR), naive Bayes (NB), support vector machine (SVM), and decision tree (DT). The MLR is analogous to the binary model and is suitable for classification without order among categories. For a K-category classification, MLR contains K−1  binary regression models. For a given input x (feature), the category with the highest probability is selected as the predicted category when operating these K−1 binary regression models. NB is a classifier based on Bayes’ theorem and the independent assumption of feature conditions. First, for a given training dataset, the joint probability distribution of the feature and prior probability model is learned based on independent assumption of feature conditions. Using the maximum-likelihood estimation function and Bayes’ theorem, the output y (classification) with the greatest posterior probability is obtained for a given input x (feature). Multinomial NB is the most commonly used model and is a bag-of-words model. The SVM classifier aims to seek the best compromise between the model complexity and the learning ability based on the statistical VC dimensions and the principle of structural risk minimization. The common kernel functions include liner, polynomial, sigmoid, and the radial basis function (RBF). The RBF kernel was used because it nonlinearly delineates samples into a higher dimensional space and ensures that the model achieves good accuracy. The penalty factor C and the kernel parameter γ are two important parameters of RBF, and these values directly affect the classification results. Therefore, we further used the grid-search method to determine the optimal C and γ. In terms of the DT algorithm, it uses decision rules to generate decision trees recursively from top to bottom. The typical classifier CART in DT was chosen for text classification in this study. The Gini coefficient was used to represent the impurity of the model.

The industrial POI samples with a total number of 16,600 were divided into a training dataset (90%) and a validation dataset (10%). For analyzing the classification accuracy of different machine learning models, the 10-fold cross-validation method was used. The classification accuracy of each model was further calculated. The abovementioned steps were conducted using Python 3.8 on the PyCharm platform. The model with the highest accuracy was selected and applied to predict the internal structure of the industrial POIs.

#### 2.2.2. Industrial Land Mapping Using Google Earth Maps

GE images were used for mapping industrial land in ArcGIS 10.2.2, ESRI, Redlands, CA, USA. The industrial POIs were superimposed on the GE images to show the precise positions of the industrial companies. To improve the efficiency of the photointerpretation process, a detailed road network and river network were also applied. The networks facilitated the industrial land digitization by providing easily recognizable geographical features.

**Figure 4:**
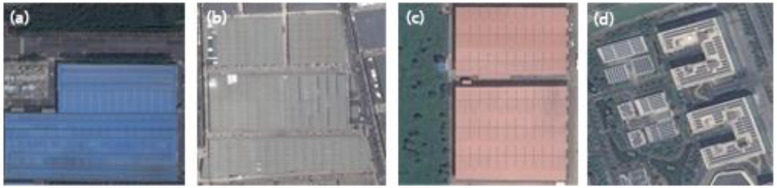
Examples of different types of industrial parks: (**a**) metal rooftops, mainly in blue; (**b**) concrete rooftops, mainly in grey; (**c**) clay rooftops, mainly in dark or red color; (**d**) concrete rooftops, which have a smaller occupied area compared with the former three types.

Industrial land has specific characteristics in GE images. The materials of industrial rooftops mainly include clay, metal, and concrete (Figure 4a–c). The traditional industrial parks have large areas; new-style industrial parks such as high-tech industrial parks are in smaller areas (Figure 4d).

When checking the industrial POIs and other POIs (i.e., residential area, commercial area, parks, etc.) from 2010 to 2015, both expansion and renewal were found. The process of industrial parcel mapping began with digitalizing the parcels in the 2010 images. The new industrial POIs from 2010 to 2015 were also superimposed, and the newly expanded industrial land was digitized in the 2015 image. In terms of the digitalization of industrial renewal, the industrial parcels that transformed into the six re-use types of transportation facilities, residential land, commercial land, green space, water body, and barren land were manually delineated with references to GE images and other POIs.

**Figure 5:**
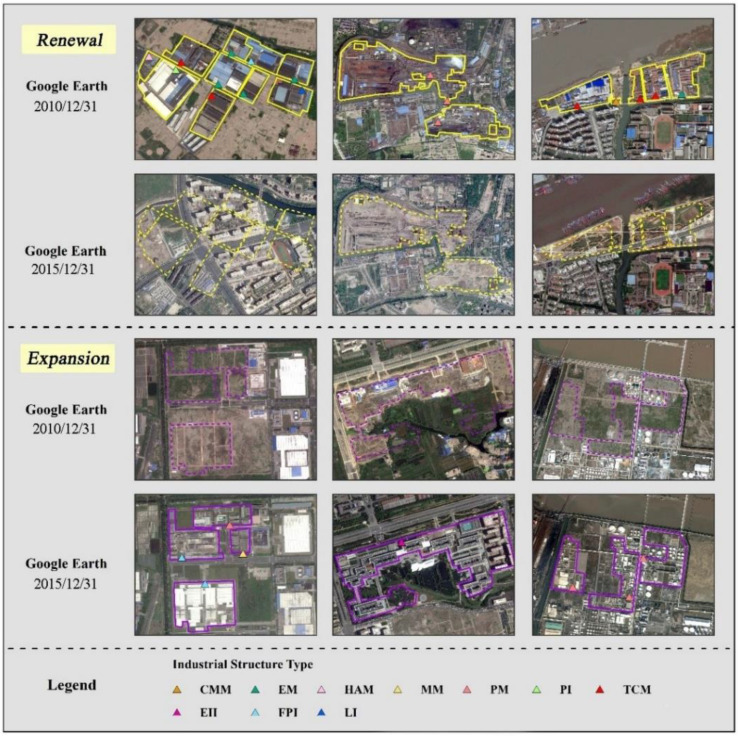
Visual examples of industrial changes (renewal or expansion) in GE images during 2010–2015.

With respect to the intra-structure of the industrial parcels, we connected the digitalized industrial land parcels with the industrial POIs through a proximity analysis, which means that the intra-structure of each parcel was equal to that of the industrial POIs. Finally, each industrial parcel was linked with its intra-structure. Figure 5 shows visual examples of industrial land and the corresponding intra-structure in Google Images.

#### 2.2.3. Spatial Measurements of Urban Industrial Land and Internal Structure

The area of each industrial land parcel in 2010 and 2015 was calculated to quantify the industry size and analyze changes in ArcGIS 10.2.2. In order to better understand the spatial patterns of urban agglomeration and industrial land as well as the uneven distribution of different industrial structures, kernel density estimation (KDE) was also adopted in ArcGIS 10.2.2. KDE is a well-proven spatial-analysis method for transforming mass geographical distributed points into a smoothly curved surface [34]. Generally, KDE exhibits a visualization of the event clusters across the study area. The visualization will represent the local probability of the emergence and extent of human industrial activities in the area. Peaks represent the occurrence of industrial hotspots, whereas low values represent industrial activities to be much weaker. Given the multivariate dataset (*x*_1_, *y*_1_), (*x*_2_, *y*_2_),…, (*x_n_*, *y_n_*), the kernel density estimator f(x,y) is computed as:

(2) $$f\left(x,y\right)=1/\left(nh^{2}\right)\sum_{i=1}^{n}K\left(d_{i}/h\right)$$

where f(x,y) is the density estimated at the location of observation (x,y), *n* is the total number of observations, *K* is the kernel function, *h* is the bandwidth parameter, and *d_i_* is the distance from the observation (x,y) to the *i*th observation. In this study, the kernel function *K* was defined as the area of each industrial land that was extracted via photointerpretation based on GE images. The selection of the bandwidth parameter *h*, which dictates the smoothness degree of an estimated density surface, is an essential task in practice. As the bandwidth *h* increases, the density surface will become smoother. If the bandwidth is too large, the key spatial fluctuations may be missed due to over-smoothing. If the bandwidth is too small, the surface will turn out to be highly fractured, thereby generating redundant wiggles [35]. Given all this, the bandwidth parameter *h* in this study was set to 2 km at the UAHB scale through tests.

## 3. Results

### 3.1. Industrial Structure Classification

**Figure 6:**
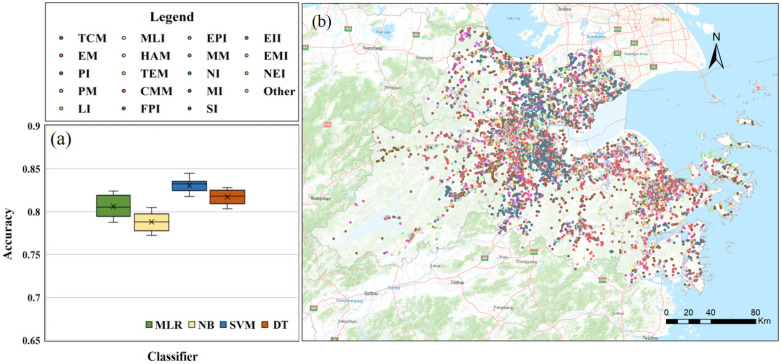
Industrial structure classification results based on industrial POIs: (**a**) accuracies of four models, (**b**) intra-structure classification result of UAHB.

To assess the classification accuracy of the different machine learning models, the training samples were divided into a calibration dataset and a validation dataset. The prediction results when using different classifiers are depicted in Figure 6. The four models performed well with an average accuracy of 83.1%. The accuracy of the SVM was the highest with a value of 84.5%, followed by DT, MLR, and NB. Then, the SVM model was applied in predicting the POIs for 2010 and 2015. We further calculated the overall accuracy (OA), producer accuracy (PA), and user accuracy (UA) of different industrial structures identified by the SVM (see Table A1). The PAs of the food-processing industry, nuclear industry, mining industry, salt industry, and the electronic information industry were over 90%, and the PAs of most industries were above 80%, which indicated that the SVM model had a good predictive ability with strong robustness. However, it is worth noting that the PA of the medical manufacturing industry was low and that several samples were confused with the construction material manufacturing, metallurgical industry, and petrochemical manufacturing, probably because the enterprise names of the medical manufacturing industry contained keywords such as chemistry, which could easily be mistaken for the other two industrial types.

### 3.2. Overall Statistics of Industrial Land and Its Internal Structure

**Figure 7:**
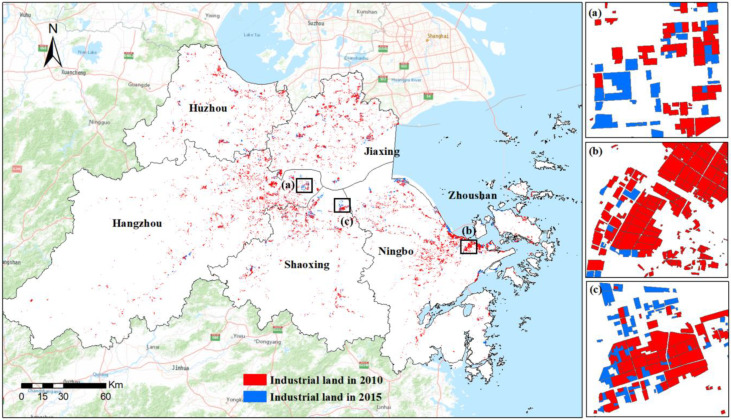
Spatial distribution of industrial land across the UAHB from 2010 to 2015: (**a**) Da jiangdong Economic Development Zone, (**b**) Ningbo Economic Development Zone, (**c**) Shangyu Economic Development Zone.

Table 3 reports the overall statistics of the identified industrial land across the UAHB at the city level. Figure 7 shows the general spatial distribution of the industrial land from 2010 to 2015. In terms of quantity, the total area of the industrial land in the UAHB increased considerably from 112,766.9 ha in 2010 to 132,124.2 ha in 2015. The industrial land changes varied among cities. During the study period, the industrial land of Ningbo increased from 32,376.8 to 38,632.4 ha, which was the largest industrial change (6255.6 ha) among the six cities. After Ningbo, Shaoxing and Jiaxing also experienced significant changes of 3617.7 ha and 3476.0 ha, respectively, and the provincial capital Hangzhou was measured to have increased by 2928.0 ha. Additionally, Huzhou and Zhoushan showed slower increases of 1900.3 and 1179.8 ha, respectively.

**Figure 8:**
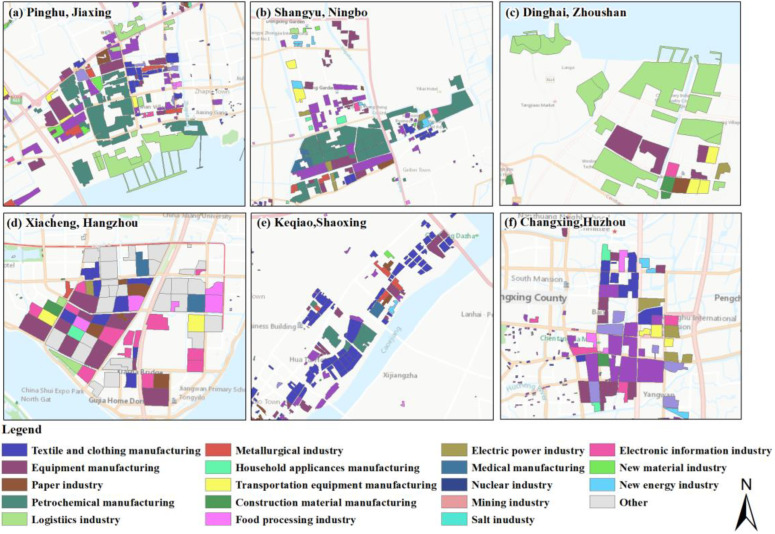
Identification of industrial intra-structure across the UAHB from 2010 to 2015.

**Figure 9:**
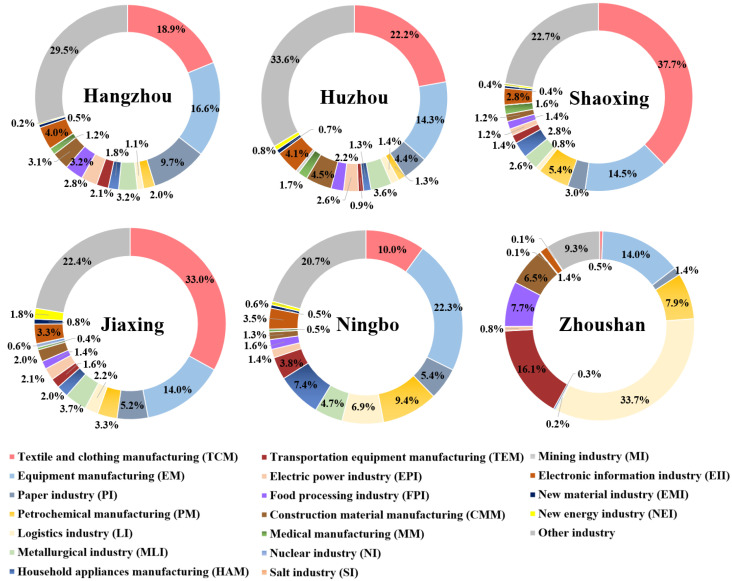
Industrial land-use proportions of different industrial structures across the UAHB in 2015.

In terms of the industrial land use structure, traditional industry occupied the largest area in the UAHB in 2015. Specifically, textile and clothing manufacturing was the most representative in terms of its percentage, followed by equipment manufacturing, the paper industry, petrochemical manufacturing, and the logistics industry. As shown in Figure 8 and Figure 9, the industrial structure pattern differed in each city. In Hangzhou, the core of the UAHB, the main industrial land structures were textile and clothing manufacturing, equipment manufacturing, and the paper industry with proportions of 18.9%, 16.6%, and 9.7%, respectively. Furthermore, as Hangzhou is a well-known city for “Internet+” development, the electronic information industry followed after those three traditional industries. In Shaoxing, approximately 37.7% of the total area of industrial land was dedicated to textile and clothing manufacturing, followed by equipment manufacturing and petrochemical manufacturing. Like Shaoxing, Jiaxing has also focused on developing textile and clothing manufacturing (33.0%), followed by equipment manufacturing and the paper industry. Ningbo, the subcore of the UAHB, mainly utilized industrial land for equipment manufacturing (22.3%), textile and clothing manufacturing (10.0%), petrochemical manufacturing (9.4%), and household appliance manufacturing (7.4%). In Zhoushan, an island city, the dominant industrial land uses were the logistics industry, transportation equipment manufacturing, and equipment manufacturing. In terms of Huzhou, the proportions of textile and clothing manufacturing, equipment manufacturing, and electric power manufacturing were relatively high at 22.2%, 14.3%, and 4.4%, respectively.

### 3.3. Characteristics of the Industrial Land Expansion and Renewal Process

**Figure 10:**
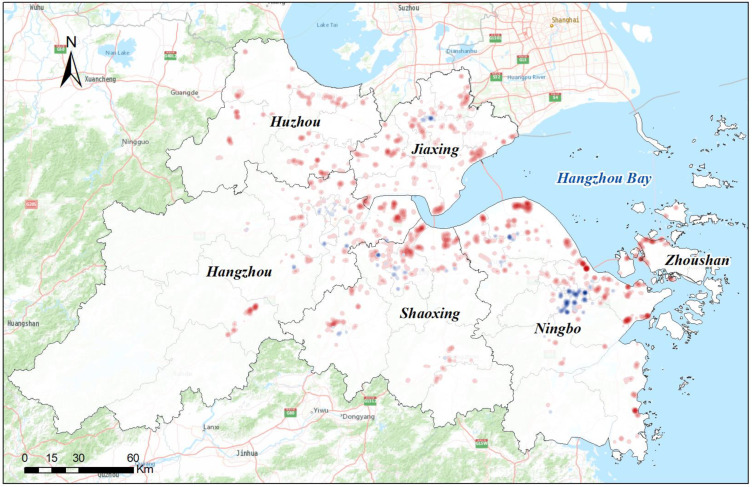
Industrial expansion and renewal intensity across the UAHB during 2010–2015. The red part (0~100%) represents expansion; the darker the red, the higher the intensity of industrial expansion.

Table 4 provides the overall statistics of the industrial land changes and intra-structure proportions from 2010 to 2015. Figure 10 shows the spatial intensity of expansion and renewal according to the kernel density estimation. It was found that a total of 2888 expansion sites (19,396.1 ha) and 182 renewal sites (581.7 ha) were in the UAHB. The renewal projects mainly occurred in the central city, which accounted for 94.2% of the total, especially in the core city of Hangzhou and subcore city of Ningbo (Figure 10). The original industrial uses of these renewal projects were mostly textile and clothing manufacturing, equipment manufacturing, and the paper industry, which accounted for 28.58%, 26.04%, and 7.98%, respectively (Table 4). Additionally, the areas with intensive expansion were mainly on the edges of cities and along the coastal zone (Figure 10). The major industrial uses of the expansion areas were equipment manufacturing, textile and clothing manufacturing, and petrochemical manufacturing, which accounted for 21.57%, 13.84%, and 10.00%, respectively (Table 4).

These results further supported the idea that reviving the existing stock and creating new engines of industrial land growth have become the two major characteristics of industrial land development in the post-industrialization stage across the UAHB. On one hand, due to the urgent need to improve urban environment and to achieve socioeconomic inclusive growth, the traditional industrial land that is randomly arranged within the central city is mostly planned to be replaced by residential, commercial, or innovational office buildings. On the other hand, as the edge of the central city has a unique location and a convenient traffic arrangement, it is more likely to attract the attention of industrial companies. In addition, the coastal zone is another potential area for industrial development. Therefore, the edge of the central city and the coastal zone became the new territory for industrial parks and open economic zones.

### 3.4. Kernel Density Estimation of Industrial Clusters in the UAHB

**Figure 11:**
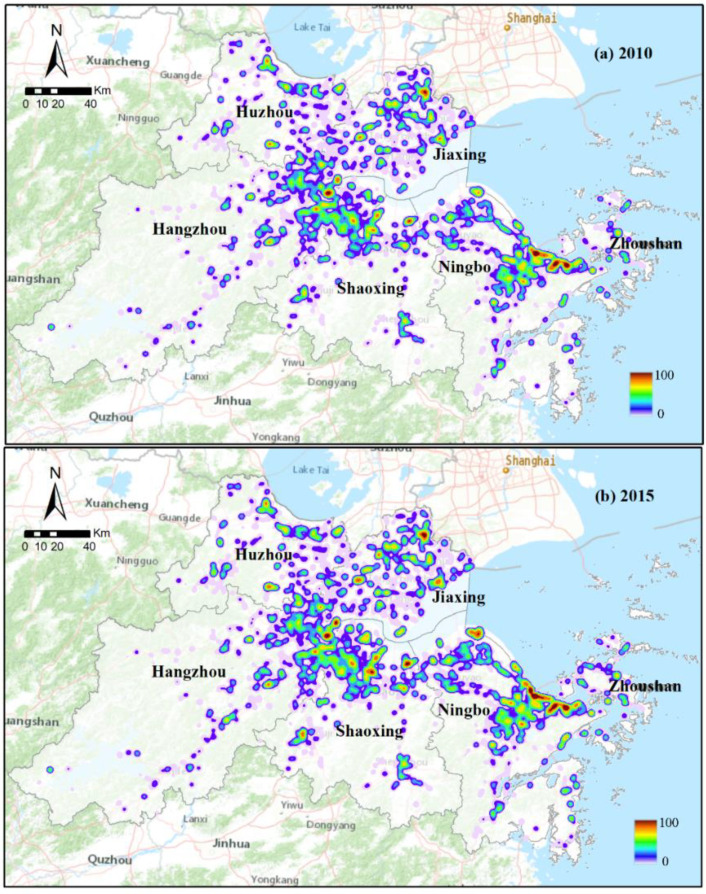
Spatial distribution of industrial cluster areas in 2010 (**a**) and 2015 (**b**).

In using the kernel density estimator, we estimated the industrial land use intensity to understand the industrial clusters in the UAHB. As illustrated in Figure 11, the industrial intensity showed an increasing trend from the central urban area to rural areas, which indicated that the industrial size was increasing and the distribution was more centralized at the urban fringes. We found that scores of industrial clusters have formed that are mainly located in eastern Hangzhou, the Huzhou border region (along Tai Lake), northern Shaoxing, coastal Jiaxing, coastal Ningbo, and northern Zhoushan mainland. Moreover, the cluster areas (Figure 11b) that are located across Hangzhou Bay tended to increase sharply in quantity and in magnitude, which suggested that the integration and union of the coastal industrial zone through transportation networks such as cross-sea bridges will be important means for the regional government to strengthen industry development in the UAHB.

**Figure 12:**
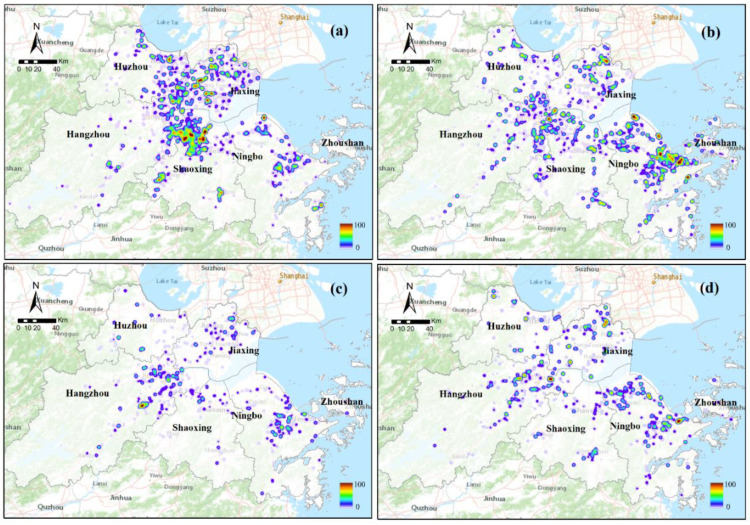
Spatial distributions of typical industrial structures in 2015: (**a**) textile and clothing manufacturing; (**b**) equipment manufacturing; (**c**) paper industry; (**d**) electronic information industry.

In terms of industrial structures, their spatial distribution varied in the UAHB. Several typical industrial structure maps are exhibited in Figure 12. For instance, textile and clothing manufacturing showed intense development in the UAHB, especially in Tongxiang, Haining county of Jiaxing, Shangyu, Yuecheng county of Shaoxing, and Nanxun county of Huzhou. Equipment manufacturing mainly occurred in the coastal zones (Beilun, Zhenhai county) of Ningbo and Xiasha and in Xiaoshan county of Hangzhou. The electronic information industry showed intense development in Yuhang and Xiaoshan counties of Hangzhou and Yinzhou county of Ningbo.

## 4. Discussion

### 4.1. Advantage and Applicability Analysis of the Methodology

In this study, we utilized both industrial POIs and GE images to map the industrial land and intra-structure at the urban-agglomeration scale. The spatial pattern of industrial land and structure composition was well reflected. Our approach had several advantages that were in contrast with the traditional survey methods. Firstly, the industrial POIs applied in this study recorded not only the location coordinates of geographical entities but also the textual information that revealed different industrial land use functions (e.g., textile and clothing manufacturing, equipment manufacturing, etc.). The industrial structure classification method that integrates NLP and ML can be considered a faster and more effective method, especially for large metropolitan areas, compared with the traditional industrial land-use field investigations. By comparing the classification results, these four models had good predictive abilities with respectively high accuracies. The SVM model performed best with an overall accuracy of 84.5% and producer accuracies for most industries of over 80%; the predicted results of the industrial classification using SVM were almost identical to the actual results. Secondly, both data sources used in our study are free and accessible as compared with other commercial very-high-resolution remote sensing images or cadastral survey data. Moreover, both the POIs and GE images are updated at regular intervals. This indicates that industrial land and its internal structure changes can be continuously monitored by keeping pace with the updating of the two datasets in the future.

Although our approach successfully monitored industrial land and intra-structure dynamics, some limitations should still be considered. First, delineating industrial parcels via manual photointerpretation of GE images is time-consuming. Therefore, further work could be undertaken toward developing a semi-automatic or fully automatic classification technique to discriminate industrial parcels in GE images [36]. Second, both Chinese text segmentation and the feature-selection procedure played significant roles in the accuracy of the industrial structure identification. In this study, the names of several companies may not have reflected their social functions, which made it difficult for us to identify the keywords for the relevant industrial category.

### 4.2. Change Dynamics

**Figure 13:**
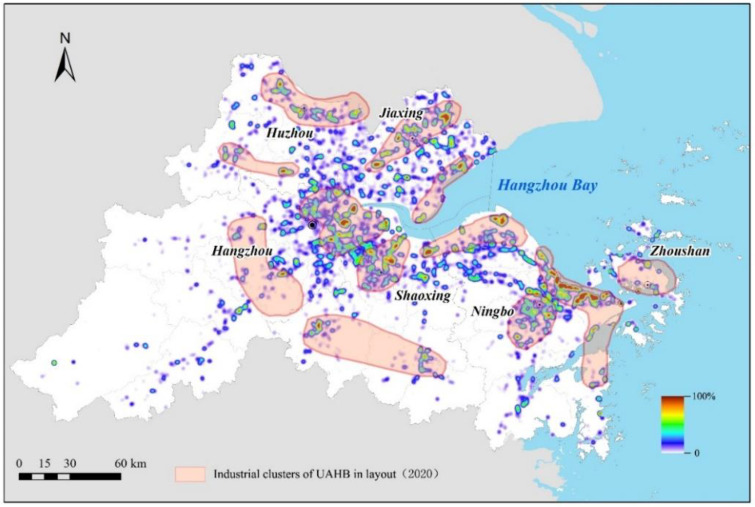
The map of UAHB’s industrial layout and spatial distribution of industrial clusters.

This study was an initial attempt to understand the spatial dynamics of industrial land and intra-structure over a five-year period across the UAHB. The spatial changes revealed some interesting findings. First of all, the industrial land was mainly concentrated at the urban fringes and industrial development zones with parcels occupied vast areas. The industrial development zones located along the coastal area of Hangzhou Bay tended to increase sharply in quantity and in magnitude during the study period. The results were consistent with other studies because they also mentioned that urban fringes and industrial development zones had higher expansion intensities of industrial land than other regions during the parallel period [37]. The phenomena of industrial land at the urban fringes and industrial development zones could be attributed to the shortage of developable land and the continuous increase in land parcel value in the urban center [10]. Many industrial enterprises were forced to seek new manufacturing space at the urban fringes in consideration of capitalized costs. Moreover, the industrial development zones of the UAHB also exhibited enormous attractiveness to industrial enterprises due to preferential terms such as sufficient land space, financial incentives, technical support, power guarantees, etc. [5] In addition, the implementation of a series of policies also played important roles in the establishment of industrial development zones [38]. As shown in Figure 13, the overall spatial distribution of industrial clusters has conformed to the layout of the UAHB industrial clusters (2020). The industrial development plans issued by government such as the Zhejiang industrial cluster zone development plan (2011–2020) have been strongly encouraged since 2010. The Zhejiang industrial cluster zone development plan (2011–2020) put forward industrial-cluster-zone construction to make room for the in-depth development of advanced industries such as new energy and new materials and equipment manufacturing. The plan also pointed out that the UAHB should speed up the transformation from a traditional lump economy to modern industrial clusters. Eight industrial cluster zones were determined as the key development zones; these include Da Jiangdong (Hangzhou), the West Science and Technology Innovation Corridor (Hangzhou), the Hangzhou Bay New Zone (Ningbo), Meishan Logistics (Ningbo), the South Taihu Lake Industrial Zone (Huzhou), the Jiaxing Industrial Zone (Jiaxing), the Coastal Shaoxing Industrial Zone (Shaoxing), and the Coastal Zhoushan Industrial Zone (Zhoushan).

Second, the industrial land-use types were mostly traditional labor-intensive industries or capital-intensive industries such as petrochemical manufacturing, equipment manufacturing, textile and clothing manufacturing, and transportation equipment manufacturing. The major industrial intra-structure varied among the different cities. The results were consistent with the development orientation and industrial economic performance of each city. The industrial structure varied between the city center and periphery. In the city center, the industrial types were mainly new industries such as the electronic information industry with a relatively high land-use efficiency, while traditional industries tended to be far away from the city center.

Thirdly, during the study period, industrial renewal was also found in parallel to industrial land expansion and structure optimization. Industrial expansion mainly occurred in the urban–rural areas and coastal areas, whereas industrial renewal mainly occurred at the city centers. Since the most incompatible manufacturing industries were forced to leave the city centers, industrial brownfields must be regenerated because the considerable amount of brownfields may lead to negative socio-psychological behaviors by citizens and introduce urban inequality and ecological degradation [39,40]. As a result, the projects of industrial renewal mostly occurred in the core area of the cities in the UAHB. These phenomena were consistent with those in other megacities of China such as Shenzhen [41] and in many dense cities abroad [42,43]. Moreover, as the government policies and plans have been essential parts of facilitating regional development in China, the projects of industrial renewal could be difficult to execute without the support of land-use policies. For example, the implementation of plans for industrial land renovation; namely, “from the secondary industry to the tertiary industry”, have contributed to improving land-use efficiency.

### 4.3. Implications for Sustainable Industrial Land Development in the Future

The regional industrial block economy is an important characteristic and the main source of economic vitality of the UAHB [44]. The UAHB has been enacting strict protection of cultivated land and the sustainable use of marine resources, which indicate that the industrial growth will be restricted within certain boundaries [45]. Now it is regarded as the key period of industrial transformation [46]. Therefore, in the future, it is essential to optimize industrial patterns and structures for quicker and better industrial development.

Firstly, we emphasize that multiple levels of industrial planning are needed for the UAHB. Irrational, redundant industrial construction and vicious competition may occur between different cities due to the administration division [47]. To strengthen the linkages of six cities and create “win–win” situations, Zhejiang’s central government should enhance the industrial macroscopic plan across the UAHB. The local government of each city must coordinate its prefecture-level plan with the overall plan of the UAHB. Based on the industrial development advantages, a scientifically based plan must be included and implemented strictly.

Secondly, we suggest that intensive utilization of industrial land should be further improved. The industrial land-use focus should be diverted to the renewal of inefficient industrial land. The projects such as “Space Exchange” and “Revitalize the Stock” should be comprehensively carried out [48]. Additionally, it is urgent to standardize and amplify the industrial land-circulation mechanism, implement policies such as the elimination of zombies or backward industrial companies, and encourage companies to develop new industry states through merger or recombination.

Thirdly, industrial parks have become the frontier for attracting foreign capital, advanced technology, and management experience due to the preferential policies and mature investment environment in China [49]. More attention should be paid to the rigorous control of companies’ entry into industrial parks. Companies that can neither meet industrial park standards nor be integrated into the industry chain must be prohibited. A comprehensive benefit-evaluation system should be created to determine whether any company fits the requirements, and the performances of companies should be under supervision. Moreover, new industries such as the Internet industry and advanced equipment manufacturing should be strongly encouraged in order to boost industry transition.

## 5. Conclusions

The emergence of geospatial big data has provided new opportunities for us to sense urban socioeconomic activities. One promising big data source—POIs—was applied in our study. POIs showed a powerful potential to characterize the intra-urban structure at a high spatial resolution and a large scale. GE images were also adopted in order to build a detailed and updated industrial-use spatial dataset. In the present study, we established a new and comprehensive framework to identify and analyze the industrial spaces and intra-structure through three steps: using NLP and ML to identify industrial structures, industrial parcel photointerpretation, and applying a KDE analysis tool. The industrial intra-structure maps were generated using a support vector machine (SVM) approach that showed an overall accuracy of 84.5%, which indicated that the SVM model performed well with a good predictive ability. The method successfully recognized the spatiotemporal pattern of the industrial space and industrial intra-structure across the UAHB.

The results revealed the higher growth of industrial parcels in the coastal UAHB and urban–rural fringes during 2010–2015. The renewal projects mainly occurred in the central city and accounted for 94.2% of the total renewal areas. After decades of mass industrial economic development, the industrial structure of each city has formed its own characteristics. Textile and clothing manufacturing occupied the most area in Shaoxing, Jiaxing, Huzhou, and Hangzhou. Adjacent cities were more likely to share similar industrial structures. Moreover, with the encouragement of industry transition across the UAHB, huge support has been given to developing new industries (e.g., the Internet industry), especially in the core city of Hangzhou and subcore city of Ningbo. The current situation and change dynamics of industries further reflect the economic progress and policy management. Our findings can serve as a fine and detailed reference for industrial land use. Our data can also be easily updated regularly. Hence, they will be helpful for government managers to supervise the extent and positions of the informal industrial expansion and the proceeding of industrial renewal projects to enact proper policies in a timely manner to achieve healthier and better industrial land utilization. Additionally, identifying the current industrial structure is a basic step in understanding the urban industry division and predicting future trends. For government managers, our findings will provide valuable references and might be useful in making proper arrangements for new industrial companies.

## Figures and Tables

**Table 1:** Industrial structure classification system.

First Class	Sub-Class
Traditional industry	Textile and clothing manufacturing (TCM)
	Equipment manufacturing (EM)
	Paper industry (PI)
	Petrochemical manufacturing (PM)
	Logistics industry (LI)
	Metallurgical industry (MLI)
	Household appliance manufacturing (HAM)
	Transportation equipment manufacturing (TEM)
	Construction material manufacturing (CMM)
	Food-processing industry (FPI)
	Electric power industry (EPI)
	Medical manufacturing (MM)
	Nuclear industry (NI)
	Mining industry (MI)
	Salt industry (SI)
New-fashioned industry	Electronic information industry (EII)
	New material industry (EMI)
	New energy industry (NEI)
Other industry	Other industry (OI)

**Table 2:** Chinese text segmentation result examples.

Number	Text Segmentation Result Example
1	Ningbo/Zhonglei/wool/textile/factory/
2	Ningbo/Xinxin/plastic/product/factory/
3	Ningbo/Fulianyue/garment/factory/
4	Chenhong/dressing/factory/
5	Shanghai/Yiyun/international/delivery/factory/
6	KaiEndi/numerical control technique/factory/
7	Ningbo/Saifu/international/delivery/factory/
8	Ningbo/newspaper/printing/factory/
9	Ningbo/Changjin/imports and exports/factory/
10	Hanlong/metallic material/technology/factory/

**Table 3:** Overall industrial dynamics of the UAHB by city.

City	2010/ha	2015/ha	2010–2015/ha
Hangzhou	26,630.8	29,558.8	2928.0
Huzhou	12,706.0	14,606.3	1900.3
Jiaxing	20,720.2	24,196.2	3476.0
Ningbo	32,376.8	38,632.4	6255.6
Shaoxing	16,925.2	20,542.9	3617.7
Zhoushan	3407.9	4587.7	1179.8
Total	112,766.9	132,124.2	19,357.3

**Table 4:** Industrial land-use proportions of expansion (renewal) projects during 2010–2015.

Industrial Structure Type	Expansion/ha	Percentage/%	Renewal/ha	Percentage/%
Textile and clothing manufacturing (TCM)	2684.38	13.84	166.25	28.58
Equipment manufacturing (EM)	4183.41	21.57	151.49	26.04
Paper industry (PI)	360.13	1.86	46.44	7.98
Petrochemical manufacturing (PM)	1939.51	10.00	12.85	2.21
Logistics industry (LI)	1210.44	6.24	2.78	0.48
Metallurgical industry (MLI)	811.35	4.18	20.87	3.59
Household appliance manufacturing (HAM)	736.47	3.80	24.24	4.17
Transportation equipment manufacturing (TEM)	1156.14	5.96	43.19	7.42
Construction material manufacturing (CMM)	341.66	1.76	23.95	4.12
Food-processing industry (FPI)	439.02	2.26	24.83	4.27
Electric power industry (EPI)	386.88	1.99	3.53	0.61
Medical manufacturing (MM)	283.82	1.46	0.73	0.12
Nuclear industry (NI)	-	-	-	-
Mining industry (MI)	-	-	-	-
Salt industry (SI)	3.12	0.02	0.13	0.02
Electronic information industry (EII)	637.96	3.29	2.83	0.49
New material industry (EMI)	374.92	1.93	-	-
New energy industry (NEI)	244.23	1.26	-	-
Other industry	3602.74	18.57	57.60	9.90
Summary	19,396.17	100.00	581.70	100.00
Percentage area in central city of total amount/%	53.30	-	94.20	-
Site number	2888	-	182	-

## Data Availability

Not applicable.

## Appendix A

**Table A1:** Confusion matrix of industrial land use identification.

	TCM	EM	PI	PM	LI	MLI	HAM	TEM	CMM	FPI	EPI	MM	NI	MI	SI	EII	EMI	NEI	OI	UA
TCM	120	2	3	2	1	0	0	0	0	3	3	4	0	0	1	0	1	1	1	84.5%
EM	3	102	1	1	2	0	5	3	3	0	3	0	0	0	0	2	2	2	0	79.1%
PI	4	0	90	2	1	2	0	0	3	1	0	3	0	0	0	0	0	0	1	84.1%
PM	0	0	2	91	1	3	3	0	5	0	0	4	0	0	0	0	1	0	0	82.7%
LI	0	0	0	0	89	1	1	4	0	1	0	2	0	0	1	0	3	5	0	83.2%
MLI	0	0	1	3	0	97	2	3	0	0	0	6	0	1	0	0	4	0	0	82.9%
HAM	2	4	0	0	0	1	105	6	3	0	7	0	0	0	0	1	0	0	1	80.8%
TEM	0	0	0	0	5	2	1	96	0	0	3	0	0	0	0	0	0	0	2	88.1%
CMM	1	2	2	1	2	1	0	1	118	0	0	7	0	0	0	0	4	0	1	84.3%
FPI	2	0	0	1	2	0	0	0	0	93	0	2	0	0	2	0	0	0	3	88.6%
EPI	0	0	0	0	3	3	2	2	0	0	116	0	0	0	0	2	0	6	0	86.6%
MM	0	0	2	4	0	2	3	1	4	0	0	98	0	0	0	2	1	0	0	83.8%
NI	0	0	0	0	0	0	0	0	0	0	0	0	8	0	0	0	0	0	0	100.0%
MI	0	0	0	0	0	3	0	0	0	0	0	1	0	46	0	0	0	0	0	92.0%
SI	0	0	0	0	0	0	0	0	0	2	0	0	0	0	36	0	0	0	0	94.7%
EII	0	3	0	0	2	3	4	0	0	0	2	1	0	0	0	102	0	1	0	86.4%
EMI	3	0	2	1	2	0	0	0	2	0	0	0	0	0	0	0	65	0	0	86.7%
NEI	0	2	1	0	1	2	1	0	0	0	0	2	0	0	0	0	2	92	3	86.8%
OI	3	2	4	1	2	2	3	1	2	0	3	2	0	0	0	0	0	1	96	78.7%
PA	87.0%	87.2%	83.3%	85.0%	78.8%	79.5%	80.8%	82.1%	84.3%	93.0%	84.7%	74.2%	100.0%	97.9%	90.0%	93.6%	78.3%	85.2%	88.9%	OA: 84.5%

PA: producer’s accuracy; UA: user’s accuracy; OA: overall accuracy.
